# Modeling the impact of school reopening on SARS-CoV-2 transmission using contact structure data from Shanghai

**DOI:** 10.1186/s12889-020-09799-8

**Published:** 2020-11-16

**Authors:** Benjamin Lee, John P. Hanley, Sarah Nowak, Jason H. T. Bates, Laurent Hébert-Dufresne

**Affiliations:** 1 Department of Pediatrics, Larner College of Medicine, University of Vermont, Burlington, VT USA; 2 Translational Global Infectious Diseases Research Center, University of Vermont, Burlington, VT USA; 3 Department of Pathology and Laboratory Medicine, Larner College of Medicine, University of Vermont, Burlington, VT USA; 4 Department of Computer Science, College of Engineering and Mathematical Sciences, University of Vermont, Burlington, VT USA; 5 Vermont Complex Systems Center, University of Vermont, Burlington, VT USA

**Keywords:** COVID-19, SARS-CoV-2, SEIR model, SIR model, Pandemic, Schools

## Abstract

**Background:**

Mathematical modeling studies have suggested that pre-emptive school closures alone have little overall impact on SARS-CoV-2 transmission, but reopening schools in the background of community contact reduction presents a unique scenario that has not been fully assessed.

**Methods:**

We adapted a previously published model using contact information from Shanghai to model school reopening under various conditions. We investigated different strategies by combining the contact patterns observed between different age groups during both baseline and “lockdown” periods. We also tested the robustness of our strategy to the assumption of lower susceptibility to infection in children under age 15 years.

**Results:**

We find that reopening schools for all children would maintain a post-intervention R_0_ < 1 up to a baseline R_0_ of approximately 3.3 provided that daily contacts among children 10–19 years are reduced to 33% of baseline. This finding was robust to various estimates of susceptibility to infection in children relative to adults (up to 50%) and to estimates of various levels of concomitant reopening in the rest of the community (up to 40%). However, full school reopening without any degree of contact reduction in the school setting returned R_0_ virtually back to baseline, highlighting the importance of mitigation measures.

**Conclusions:**

These results, based on contact structure data from Shanghai, suggest that schools can reopen with proper precautions during conditions of extreme contact reduction and during conditions of reasonable levels of reopening in the rest of the community.

## Background

The COVID-19 pandemic presents an unprecedented global public health challenge. A crucial issue that remains unresolved is the role of children in SARS-CoV-2 transmission and the impact of schools on epidemic spread. Available evidence suggest that children, particularly children < 10 years, are less susceptible to SARS-CoV-2 infection [1–4] and rarely transmit infection to adults or schoolmates [5]. However, guided chiefly by prior models of pandemic influenza, which appears to be much more transmissible among children, school closures have been a nearly universal component of pandemic response [6]. Some mathematical modeling studies suggest that school closures alone have limited effects on SARS-CoV-2 transmission [1, 7], which has been interpreted by some to suggest that little harm can follow from school reopening. Reopening schools in the setting of strict community-wide physical distancing, however, reintroduces a mode of disease transmission that is far less redundant than typical community social networks, and therefore possibly much more important. Therefore, we utilized a previously published dataset of contact structures from Shanghai pre- and post-pandemic “lockdown” to model disease transmission under various school reopening scenarios.

## Methods

We consider an age-stratified model where individuals are distinguished by their age, binned in groups of 5 years (e.g. 0–4 years, 5–9 years, and so on up to 65+ years). Distinguishing different age classes allows us to model the age-specific contact structure due to schools, households, and other social structures. These contact structures will be informed by those collected in Shanghai before and after “lockdown,” as reported by Zhang et al. [1]. Moreover, the age classes provide a simple way to account for heterogeneous susceptibility. While we relax this assumption, we initially follow Ref. [1] and set the susceptibility of children 0–14 years to be 34% of adult susceptibility and the susceptibility of individuals 65+ years to be 144% that of adults 20–65 years.

We then focus our study on the basic reproduction number R_0_ of a disease model that incorporates this age and contact structure with a given transmission rate (set to the susceptibility of adults and modulated for other age classes) *β,* and a uniform recovery rate for all age classes *γ*. Using baseline contact patterns and a fixed set of epidemiological parameters, we can calculate what we call the *baseline R*_*0*_ of the epidemic. Then, using a modified set of contact patterns that reflect specific interventions both within and outside of schools, we get a *post-intervention R*_*0*_, not to be confused with the effective reproduction number (often described as R_E_ or R_t_). Importantly, post-intervention R_0_ will always be proportional to the baseline R_0_, meaning that any set of parameters that produce the same R_0_ will produce the same post-intervention R_0_ under a given intervention. There is therefore no need to sweep both recovery and transmission rates but only one of them in order to explore a wide range of baseline R_0_ values. In our codes, available online, we choose to fix γ = 1/5.1 days as used in the original model [1, 8] and we vary β in order to vary the baseline R_0_. Importantly, note that if we consider short-term dynamics and therefore ignore demographics (e.g. birth and death rate of the population), the reproduction number of our disease model will be proportional to *β/γ* regardless of whether we implement susceptible-infectious-recovered (SIR), or susceptible-exposed-infectious-recovered (SEIR), or any other classic model [9]. Hence, we do not need to pick a particular disease model to calculate R_0_. We do, however, need to take the contact structure across age-classes into account. Let us call *K* the matrix whose elements are *K*_*i,j*_ *= σ*_*i*_*M*_*ij*_ where *i* and *j* are age classes, *σ*_*i*_ is the susceptibility of class *i*, and *M*_*ij*_ is the frequency of contacts with class *j* for an individual in class *i*. Following Ref. [10], R_0_ is given by

$$ {\mathrm{R}}_0=\kern0.5em \frac{\beta }{\gamma}\lambda (K) $$

where *λ(K)* denotes the largest eigenvalue of *K*.

Even more important is the fact that this definition of R_0_ is not only valid for SIR or SEIR models using the same age-structure and heterogeneous susceptibility, it is also valid for stochastic models based on branching processes set by the contact matrix and transmission rate. See, for example, Ref. [11] for a derivation of this equivalence. The previous definition of R_0_ is therefore applicable to a large range of epidemic models parameterized by the transmission rate *β*, the recovery rate *γ*, the heterogeneous susceptibility *σ* and the contact matrix *M*. We can then keep all parameters fixed and modify only elements of the contact matrix *M* that correspond to different school reopening scenarios. In so doing, and by focusing on R_0_, we are studying the impact of school reopening while relying on as few model-specific assumptions and mechanisms as possible.

From this basic model, we look at the impact of two key variables, the contact matrix M and the heterogeneous susceptibility *σ*. First, we combined the observed “lockdown” contact matrix with different weighted blocks of the baseline contact matrix to mimic different scenarios for school reopening and background interventions. For example, since the model stratifies the population in bins of 5 years, we can model school reopening for children < 10 years by using baseline values for the 2 × 2 block of the contact matrix corresponding to interactions between children 0–4 years with one another, between children 0–4 years with those 5–9 years, between children 5–9 years with those 0–4 years, and between children 5–9 years with one another. Other values can also be weighted to a fraction of the true baseline value to mimic partial reopening or intervention conditions. Second, we relaxed assumptions of heterogeneous susceptibility across age groups. Mainly, Zhang et al. estimated a relative susceptibility of roughly 34% for children < 15 years compared to adults [1]. We relaxed this assumption by increasing the relative susceptibility of children to different values (34, 40, 45, 50, 60%) while leaving older populations unchanged.

This model is available at https://github.com/LaurentHebert/school-reopening.

## Results

**Fig. 1:**
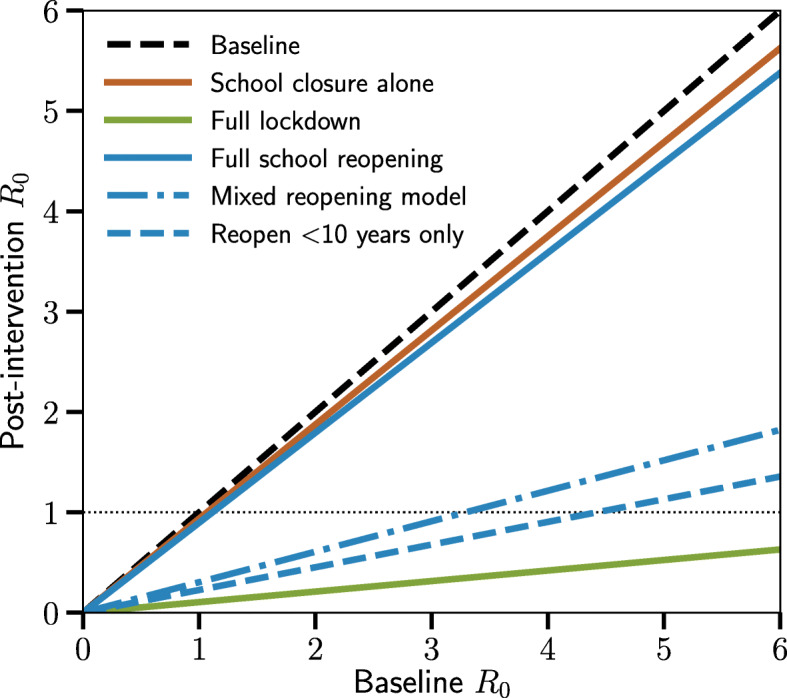
Effects of school reopening during community “lockdown.” Post-intervention R_0_ as a function of baseline R_0_ under various conditions are shown. Dashed black line: Baseline, represents all contact patterns pre-pandemic. Solid orange line: School closure alone, represents community pre-pandemic contact patterns but with contacts among children 0–19 years removed to simulate full school closure. Solid green line: Full “lockdown,” represents full contact suppression during pandemic conditions. Solid blue line: Full school reopening, represents full “lockdown” conditions but with re-incorporation of all contacts among children 0–19 years according to baseline contact patterns to simulate return to full school attendance. Interrupted blue line: Mixed reopening model, simulates the effect of re-incorporating full contact patterns for children 0–9 years with reduction in contacts in children 10–19 years to 33% of baseline. Dashed blue line: Reopen < 10 years only, simulates the effect of re-incorporating baseline contact patterns for children 0–9 years only

When no measures are taken to reduce R_0_, baseline R_0_ and post-intervention R_0_ are identical (Fig. 1, dashed black line). School closure alone has minimal effect (Fig. 1, orange line) because disease continues to spread via alternate social contacts in the community. Full “lockdown,” in contrast, has a major effect (Fig. 1, solid green line) because it severs most social contacts. Therefore, to simulate the effect of school reopening against this background, we reincorporated baseline contact patterns for children (aged 0–19 years) into the full “lockdown” model, using the same underlying assumptions for contact patterns and reduced susceptibility to infection by age as reported for Shanghai during outbreak conditions [1]. This shows a dramatic effect (Fig. 1, solid blue line): reopening schools without measures to reduce daily contacts would return transmission levels virtually to baseline despite strict physical distancing in the rest of the community, and thus would be highly inadvisable. The fact that school closures alone have little impact does not imply that school reopening during a “lockdown” will similarly have little impact.

We then assessed various conditions for school reopening to estimate impacts on post-intervention R_0_, including implementation of measures to reduce contacts among children. We find that reopening schools for children < 10 years, even without reduction in daily contacts, is predicted to maintain post-intervention R_0_ < 1 (and suppress virus transmission) up to a baseline R_0_ of ~ 4.5 (Fig. 1, dashed blue line). The addition of school reopening with reduction in daily contacts among children aged 10–19 years to 33% of baseline is predicted to keep post-intervention R_0_ < 1 up to a baseline R_0_ of ~ 3.3 (Fig. 1, interrupted blue line). These results suggest that interventions to reduce the number of contacts at school, with an emphasis on children aged 10–19 years, is a potentially viable approach to school reopening even during periods of significant baseline community transmission of SARS-CoV-2 while strict contact suppression is maintained in the rest of the community. We find that reopening schools to children < 10 years would have the least impact on disease transmission, even when we assumed that these children would be unable to adhere to interventions to reduce their effective number of daily contacts.

**Fig. 2:**
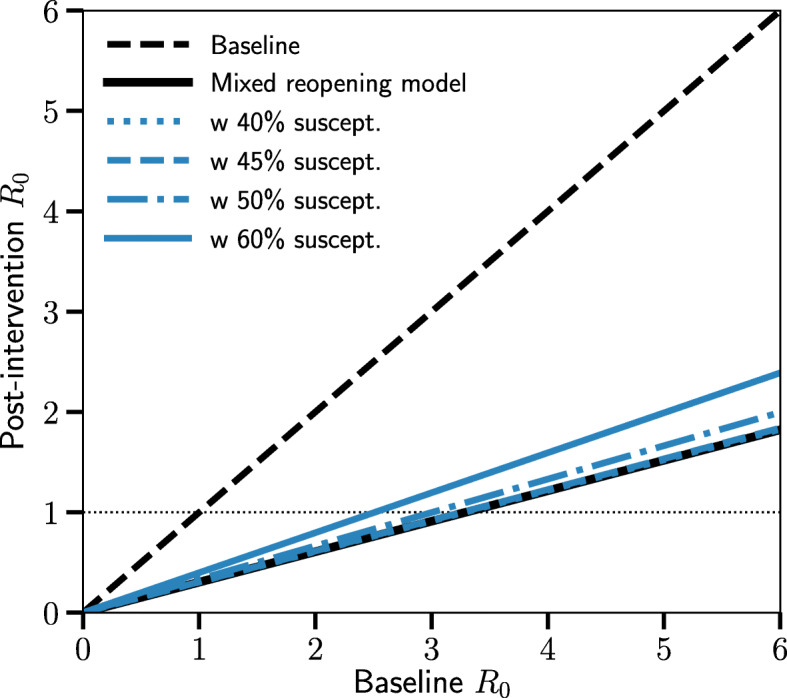
Effects of school reopening based on differing rates of susceptibility to SARS-CoV-2 infection in children relative to adults. Post-intervention R_0_ as a function of baseline R_0_ under various estimates of susceptibility to SARS-CoV-2 infection in children < 15 years are shown. Dashed black line: Baseline, represents all contact patterns pre-pandemic. Solid black line: Mixed reopening model, simulates the effect of re-incorporating full contact patterns for children 0–9 years with reduction in contacts in children 10–19 years to 33% of baseline. Starting from this condition, blue lines represent a range of estimates of susceptibility to SARS-CoV-2 infection in children relative to adults: 40% (dotted blue line), 45% (dashed blue line), 50% (interrupted blue line), and 60% (solid blue line)

The feasibility of these interventions rely in part on the limited contacts between children and older populations, but also on estimates of their lower susceptibility to SARS-CoV-2. Given that the model developed by Zhang et al. estimated a relative susceptibility of roughly 34% for children under 15 years compared to adults [1], we next looked at the robustness of our results to varying estimates of susceptibility (Fig. 2). We increased the relative susceptibility of children up to 60%, and found that our suggested reopening model remained quite robust to changes in virus susceptibility among children. In particular, the idea of full reopening for children under 10 years with contact reduction for children 10–19 years remained feasible up to a baseline R_0_ of ~ 3, even when relative susceptibility of children was estimated at 50% that of adults, itself a 50% increase compared to the original model estimates and consistent with other recent estimates [12].

**Fig. 3:**
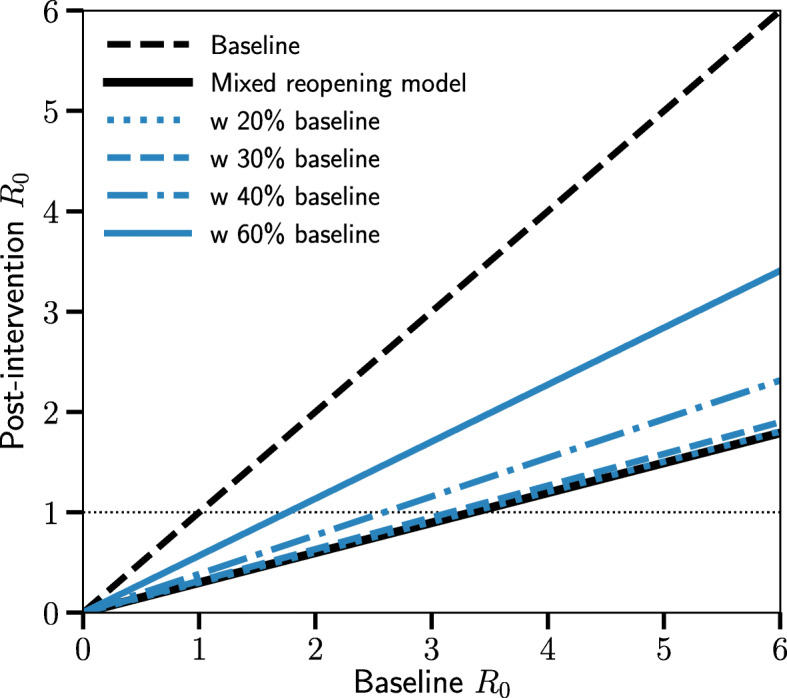
Effects of school reopening along with community reopening. Post-intervention R_0_ as a function of baseline R_0_ under various conditions are shown. Dashed black line: Baseline, represents all contact patterns pre-pandemic. Solid black line: Mixed reopening model, simulates the effect of re-incorporating full contact patterns for children 0–9 years with reduction in contacts in children 10–19 years to 33% of baseline. Starting from this condition, blue lines represent the effects of restoration of contact frequency in the rest of the community (i.e. community reopening) to 20% of baseline (dotted blue line), 30% of baseline (dashed blue line), 40% of baseline (interrupted blue line), or 60% of baseline (solid blue line)

Recognizing, however, that school reopenings would generally occur alongside other relaxations of community restrictions, we then looked at the robustness of this model in the context of gradual increases in the frequency of contacts for the rest of the community (Fig. 3). We find that return of contact frequency to 20% (Fig. 3, dotted blue line) and 30% (Fig. 3, dashed blue line) of pre-pandemic baseline among all other community members has virtually no additional impact on transmission. At 40% of baseline, post-intervention R_0_ remains suppressed < 1 up to a baseline R_0_ of ~ 2.5, and at 60% of baseline, post-intervention R_0_ remains suppressed < 1 up to a baseline R_0_ of slightly less than 2. These results suggest that even with relaxations in contact reduction measures in the rest of the community, school reopening remains feasible with reasonable measures to reduce contact frequency in the school setting.

## Discussion

In a model of SARS-CoV-2 transmission utilizing contact patterns obtained from Shanghai [1], we find that while school closure alone does not have a major impact on transmission, full school reopening during a “lockdown” without mitigation measures in the school setting can return transmission to its baseline value. That being said, we find that careful school reopening can proceed while maintaining post-intervention R_0_ < 1 under a wide range of both baseline R_0_ levels and estimates of susceptibility to infection in children, provided that appropriate measures are taken in the school and community settings to reduce the number of daily contacts among both children and school and community members. We find that younger children < 10 years have the least impact on disease transmission, and greatest priority for mitigation strategies in the school setting should therefore focus on children 10–19 years of age.

This model suggests that having open schools can and should be considered, along with mitigation strategies in both schools and the community, even during periods of SARS-CoV-2 community transmission. We recognize that the R_0_ value alone should not be used as the sole criterion for formulating a comprehensive public health strategy. Even at R_0_ < 1, various other factors (such as overall community prevalence) can profoundly impact both the rate and extent of disease transmission in the community, which also require careful consideration in school reopening decisions. Nevertheless, depending on local conditions, school closures need not be considered a necessary component of community-level SARS-CoV-2 public health response, particularly considering the profound adverse consequences of prolonged school closures on the educational, emotional, and psychosocial development of children [13, 14]. This is particularly applicable to school reopening for children < 10 years old (approximately grade 5 and lower), as has now been strongly endorsed in the United States by the American Academy of Pediatrics and the National Academies of Science, Engineering, and Medicine [15, 16]. In this age group, our model suggests that full reopening would have very minimal effect on R_0_, even without reduced contact frequencies among children in this age bracket. Any school reopening scenario may require a trade-off of maintaining more severe restrictions in other community arenas (e.g. limiting reopening of indoor spaces in bars and restaurants) in order to keep community contact frequency below the targets necessary to allow for school reopening suggested by this model. It is important to remember that our results only apply to overall community transmission and do not address individual health outcomes or the possibility and effects of transmission events within individual schools.

In this model, contact suppression was calculated as a percentage of baseline, pre-pandemic contact patterns. The definition of contact used was very broad, being either 1) two-way conversation involving three or more words in the physical presence of another person (conversational contact), or 2) a direct physical contact (e.g., a handshake, hug, kiss or performing contact sports) [1]. Sensitivity analysis indicated that for Shanghai, restricting contacts to those of at least five-minute duration (thus eliminating purely incidental, casual contact) resulted in similar results as when all contacts were considered [1]. The current definition of close contact used by the Centers for Disease Control and Prevention is even more restrictive: at least 15 min within 6 ft (approximately 2 m) of a person with confirmed infection [17].

Reducing the number of effective daily contacts could occur via complete removal of a specific proportion of typical contacts, which would be more likely during conditions of full” lockdown” or during more restrictive limitations on community movement. In Shanghai during” lockdown”, for example, contacts among school-aged children were reduced to almost zero [1]. This may not be a reasonable expectation for other regions. A comprehensive model from the United Kingdom assessing full community social distancing, for example, estimated this to represent at best a 75% reduction in all contacts outside of school, workplaces, and the household, but would likely be associated with increased household contact frequencies [7].

However, it is also very likely that other non-pharmaceutical interventions, particularly cloth facial coverings and emphasis on physical distancing, would also reduce risk of transmission during any individual encounter and convert many “at-risk” contacts into lower-risk contacts [18]. These interventions were not included as discrete variables in this model, but it would be reasonable to assume that contacts that occur with both participants wearing a cloth facial covering, at increased physical distance, or both would contribute to the percent reduction in “at-risk” contacts that we modeled due to their functional effects in terms of reduced transmission risk rather than complete contact removal. Additional strategies for reducing the frequency of close contacts within school settings have been proposed by the World Health Organization and the Centers for Disease Control and Prevention, such as eliminating large group activities, reducing student movement, and allowing for a mixture of in-class and remote learning to reduce classroom size and density [19, 20]. Scheduled hand hygiene and frequent disinfection of common surfaces would also reduce potential transmission.

Another important consideration is that this model did not consider the potential for reduced transmissibility from children to other contacts. Multiple studies now suggest that children, particularly younger children, are far less likely to transmit SARS-CoV-2 to other contacts, even within households, where the intensity of contact is arguably highest [5, 21–24]. Additional work also suggests that transmission of infection from younger children within the school setting is rare [25–29]. Therefore, the potential impact of school reopening may in fact be overestimated, as we assumed equal likelihood for transmission of virus from infected children of any age as from adults.

The range of baseline R_0_ values we identified as capable of permitting various school reopening scenarios is within the range of estimated values observed at many locations. Because school reopening decisions should depend on local, rather than regional or country-wide trends, it is most useful to assess R_0_ in this context whenever possible at as local of a scale as can be reasonably estimated. In Shanghai, R_0_ has been estimated at 3.31 up to February 16, 2020, spanning both pre- and early post-“lockdown” conditions [30], and up to 3.63 during pre-“lockdown” conditions [31]. Recent estimates from the United States suggest that R_0_ in six major metropolitan cities (Boston, Chicago, Los Angeles, Miami, New Orleans, New York City) in March 2020, near the initial peak of the outbreak in these regions, ranged from 2.43 (95% confidence interval (CI), 2.05–2.82) in New Orleans to 3.18 (95% CI 2.57–3.79) in Boston, and fell significantly thereafter once mitigation strategies were enacted [32]. If so, this suggests our model would have been applicable to both Shanghai and these selected US regions given these R_0_ estimates, particularly once community mitigation strategies had been enacted. An important caveat is that this would only hold true if the community contact structures in these US cities were sufficiently similar to that of Shanghai, which may not necessarily be the case, underscoring the need for local data to provide the most informed model predictions.

There are several limitations to these findings. Notably, as discussed previously, the baseline and outbreak contact patterns utilized in this model, which used data from Shanghai, may not be generalizable to all settings due to underlying differences in social contact networks and the achievable magnitude of contact suppression during mandated physical distancing. Therefore, similar approaches using contact structure data from other locations require further investigation. This model would not apply to college or university settings (based on an upper age limit of 19), nor to boarding schools. Based on a preponderance of current evidence, this model assumes that children are less susceptible to infection; since school closures were typically implemented along with community physical distancing mandates [6], this observation could be an artifact of limiting child contacts to within households early in the pandemic rather than a true biological difference. If children prove to be equally susceptible to infection, this model may significantly underestimate the impact of school reopening, although this may be mitigated by the effect of universal masking and increased physical distancing within the school environment. Therefore, school reopening would require flexibility to rapidly adapt to changing local conditions, along with capacity for aggressive testing and contact tracing of infected children and their families; because infected children generally have mild symptoms [33], school-associated outbreaks might present with clusters of illness in parents or household contacts.

Another important caveat of our study is that we focus solely on the spread of SARS-CoV-2 at the community level and not on outcomes for either infected children or their contacts in whom secondary infections may result (such as teachers), including the potential for mortality or other severe outcomes that may be heavily associated with specific risk factors, including age. The effects of infection in some children may also be more severe than previously appreciated, due to development in a small minority of infected children of a novel and serious multisystem inflammatory syndrome associated with COVID-19 (MIS-C), even though this condition appears to be very rare, estimated at 2/100,000 children in New York State [34, 35]. Nevertheless, prolonged school closures also come with serious risk of harm to children and families. During the pandemic, which has been universally associated with prolonged school closures in most settings, numerous reports indicate increasing rates of mental health problems, food insecurity, loss of health care coverage, and concern for increases in physical, emotional, and sexual abuse as a result of home confinement, in addition to loss of educational attainment [14, 36–40]. The individual risks to children, teachers, and families as a result of potential COVID-19 illness associated with school exposure must be balanced against these profound adverse effects which are certain to continue in the setting of prolonged school closures.

We also assume homogeneous transmission and contact patterns within a given age-class, without attempting to account for pre-existing biological or behavioral heterogeneity that can exist among individuals of the same age. The model therefore averages over the risks of super-spreading events and variable levels of adherence to public health recommendations for individuals within the same age class. While there exist network models to account for individual differences [41] these are much harder to parameterize with available data on contact patterns. Despite these limitations, the use of a simple model such as this that focuses on community-level transmission rates can nevertheless be a powerful tool for examining the larger-scale effects of significant alterations to community structure (e.g. school reopening).

Similarly, we looked at the impact of school reopenings without accounting for possible secondary changes in behavior among parents and other contacts. It is possible that school reopenings could lead to behavioral changes that would increase transmission risks in the community outside the school setting (for example, by relaxing attitudes or concerns regarding physical distancing or maximum group sizes). This might have two major unintended consequences, both detrimental. First, it could lead to increased viral transmission overall and loss of epidemic control. Second, this increase in transmission might erroneously be attributed to school reopenings themselves, prompting re-closures (and their attendant educational, economic, and societal harms), which would then be minimally effective at curtailing further transmission. Therefore, school reopenings necessitate careful public health messaging to reinforce the need for ongoing community-wide measures and to place the potential impact of school reopenings into proper context, to limit viral transmission.

## Conclusions

Schools can be reopened in the setting of ongoing SARS-CoV-2 community transmission provided appropriate and reasonable precautions are maintained to reduce the background rate of daily contacts in the community along with reductions in daily social contacts among children in the school setting. The impacts of prolonged school closure on child health, development, and education may be profound, and for most children and families, particularly younger children with working parents, remote learning has been an alarmingly poor substitute for the classroom [14, 40, 42]. We argue for a paradigm that prioritizes open schools, rather than viewing school closures as necessary adjuncts to other community-level interventions [6, 43], and that approaches based on influenza suppression may be ill-suited for the current pandemic given the clear differences between influenza and SARS-CoV-2, particularly regarding their effects on children. Strategies for reopening schools can be guided by mathematical modeling approaches, particularly wherever contact data are available to generate local estimates to inform public health policy.

## Data Availability

All data for this model are publicly available at https://github.com/LaurentHebert/school-reopening. The original data are publicly available within the main body or supplementary files of the original work by Zhang and colleagues [1], which was published under a Creative Commons Attribution 4.0 (CC BY 4.0) license, and were also posted by the authors for public availability at https://zenodo.org/record/3775672.

## Abbreviations

SEIR: Susceptible-exposed-infectious-recovered
SIR: Susceptible-infectious-recovered
R_0_: Basic reproduction number
